# Description and outcomes of Afro-Caribbean children treated for multisystem inflammatory syndrome in the French West Indies

**DOI:** 10.1016/j.heliyon.2023.e22642

**Published:** 2023-11-19

**Authors:** Charlène Grabot, Mélanie Brard, Daphnée Hilaire, Moustapha Drame, Gwladys Nadia Gbaguidi, Narcisse Elenga, Saskia Tuttle, Yves Hatchuel, Michaël Levy, Olivier Flechelles, Arthur Felix

**Affiliations:** a Pediatric Intensive Care Unit, University Hospital of Martinique, Fort-de-France, France; b Antilles-Guyane M3C Pediatric Cardiology Center, University Hospital of Martinique, Fort-de-France, Martinique, France; c Department of Pediatrics, Guadeloupe University Hospital, Pointe-à-Pitre, France; d Department of Clinical Research and Innovation, Martinique University Hospital, Fort-de-France, France; e Scientific Researcher (EMERGEN Referent), Santé publique France Antilles, Guyane, France; f Department of Pediatrics, Andrée Rosemon Hospital, Cayenne, France; g Department of General Pediatrics, Competence Center for Rheumatic, Autoimmune and Systemic diseases in Children (RAISE) Antilles-Guyane, Martinique University Hospital, Fort-de France, France; h Pediatric Intensive Care Unit, University Hospital Robert-Debré, Paris Cité University, Paris, France; i Department of Pediatrics, Reference Center for RAISE, University Hospital Robert-Debré, Paris, France

**Keywords:** Multisystem inflammatory syndrome in children, SARS-CoV-2, Child, Adolescent, Acute heart failure, Echocardiography, Pediatric intensive care unit

## Abstract

**Introduction:**

Several studies have reported a higher frequency and greater morbidity and mortality of multisystem inflammatory syndrome in children (MIS-C) of black African descent.

**Objectives:**

We aimed to describe the clinical, laboratory and echocardiographic characteristics as well as outcomes of children with MIS-C requiring admission to a pediatric intensive care unit (PICU) in the French West Indies (FWI), where the majority of the population is Afro-Caribbean.

**Methods:**

Ambidirectional observational cohort study between April 1, 2020 and August 31, 2022. Children (age ≤18 years) with MIS-C and organ failure were included. Every patient was monitored and treated following the same protocol, with repeated biological tests, echocardiography, intravenous steroids and polyvalent immunoglobulins. The primary outcomes were clinical, laboratory and echocardiography characteristics.

**Results:**

Forty children (median age 7 years, range: 5–11) were included. The majority (77 %) were included prospectively. Thirty-five (87 %) had gastrointestinal symptoms, 30 (75 %) presented initial heart failure (with persisting diastolic dysfunction at day 7) and 18 (45 %) had pericarditis. Sixteen (40 %) were in cardiogenic shock and required inotropic support. Median duration of inotropic support and hospitalization in PICU were respectively 4 and 5 days. The evolution curves of the inflammatory variables matched after treatment. The clinical outcomes were favorable. The Delta variant was associated with the highest incidence of MIS-C.

**Conclusion:**

This is the first description of MIS-C course among children of Afro-Caribbean descent. The outcomes were good, without any death or cardiac sequelae. Our work does not support an ethnic susceptibility for severity of MIS-C in Afro-Caribbean population.

## 1 Introduction

Starting in April 2020, following the coronavirus disease 2019 (COVID-19) pandemic, reports emerged from Europe and North America describing clusters of children and adolescents requiring admission to pediatric intensive care units (PICU), with a multisystem inflammatory condition resembling Kawasaki disease (KD) or toxic shock syndrome [[1], [2], [3]]. Children typically presented onset of symptoms around four weeks after COVID-19 infection [1,4].

The World Health Organization (WHO) developed case definitions, and the disorder has now been named multisystem inflammatory syndrome in children (MIS-C), associated with COVID-19 [4]. MIS-C can affect multiple organs and can progress rapidly, with heart failure and cardiogenic shock requiring admission to a PICU [[5], [6], [7]]. The severity of the disease requires multidisciplinary pediatric care, including internists, immunologists, cardiologists and intensivists [8]. Although the disease has now been well characterized, the clinical picture of the disease may vary depending on the geographic and ethnical ground. Several studies during the first wave reported that among MIS-C patients, the proportion of black children was higher that of than other ethnic groups [1,7,9]. Martinique University Hospital is the only PICU in the French West Indies (FWI), and is also a center of excellence for pediatric cardiology and pediatric internal medicine, certified by the French Ministry of Health. Although there are no official statistics on ethnicity, because collecting ethnic origin is prohibited by French law, a large majority (>90 %) of patients in the FWI are of black African descent [10].

The purpose of this multicenter study was thus to provide the first description of the day-by-day clinical, biological, echocardiographic characteristics and outcomes of patients with MIS-C hospitalized in the FWI among children of Afro-Caribbean descent.

## 2 Methods

### 2.1 Study design

We performed an ambidirectional, multicenter, observational study, in the University Hospital in Martinique, the University Hospital of Guadeloupe and Andree Rosemon Hospital in French Guiana (together totaling around 200 to 250 admissions to PICU per year). Every child meeting the WHO definition for MIS-C [4] admitted to PICU between April 1, 2020, and August 31, 2022 in any of the three centers was included.

### 2.2 Epidemiological data

The data about epidemic waves and SARS-CoV-2 variants were provided by Santé Publique France (SPF) Antilles. Data on SARS-CoV-2 variants in the FWI were extracted from the GISAID database to compare their evolution with the dynamics of MIS-C cases. Only polymerase chain reaction tests (PCR) were taken into consideration to determine the variants.

### 2.3 Data collection

The first part of the data collection was retrospective (from April 1, 2020 to August 1, 2021), and thereafter, data were collected prospectively until August 31, 2022. For the retrospective part, data was retrieved from follow-up and hospitalization reports available in the patients’ medical files. Hospital units that could refer patients to PICU included general pediatric units and emergency departments. Patients could also be referred from regional competence centers (pediatric cardiology and pediatric internal medicine). All patients had infectious explorations to rule out bacterial infection, including repeated blood cultures, urinary cultures, chest X-ray and stool culture.

### 2.4 Outcome definitions

Heart failure was defined as hypotension requiring vascular filling, altered cardiac function on ultrasound or increased cardiac enzymes. A complete biological workup was performed daily from admission, including blood count, coagulation parameters, liver function, inflammatory syndrome, cardiac enzymes (BNP, troponin) and lactates. Transthoracic echocardiography was performed by a pediatric cardiologist on the day of admission (day 0), and at day 3 and day 7 of hospitalization.

The pediatric cardiologist estimated left ventricular function with the left ventricular ejection fraction (LVEF, in percent) by the Teichholz method, and LV strain by 2D ultrasound. Systolic dysfunction was defined as mild (45 %≤LVEF<60 %), moderate (30 % ≤ LVEF ≤44 %) and severe (30 % < LVEF) and strain was considered if > −20 %. The left diastolic dysfunction was evaluated by isovolumic relaxation time (IVRT), IVRT<90 m s was considered abnormal; as well as an estimation of the vascular filling (using early to late diastolic transmitral flow velocity (E/A) and E to early diastolic mitral annular tissue velocity (E/e')). Right ventricular function was measured with tricuspid annular plane systolic excursion (TAPSE; considered normal for values > −2 standard deviation in age) and Tricuspid S wave (normal >9.5 cm/s).

### 2.5 Treatment protocol

All patients were treated according to a standardized departmental protocol (Supplementary Fig. 1). If the patient had a cardiac dysfunction or cardiogenic shock, treatment comprised high-dose steroids, i.e. methylprednisolone 10 mg/kg/day for 3 days and polyvalent immunoglobulins (IVIg) 1 g/kg/day intravenously for 2 days (with a minimum of 24 h of infusion to avoid fluid overload). Corticosteroid therapy tapering to 1 mg/kg/12 h began after 36 h if the patient was non-febrile. In case of persistent hyperthermia 48 h after completion of IVIg, a second session of IVIg and biological therapy (with the interleukin-1 receptor antagonist anakinra) was discussed. Patient management required daily clinical meetings with pediatric intensivists, internists and cardiologists. All clinical, biological and ultrasound data are recorded daily in a prospective manner, and thus, these data were available in the medical files for the patients who were identified in the retrospective part of this study (i.e. from April 1, 2020 to August 1, 2021).

#### 2.5.1 Ethical considerations

The study was approved by the Institutional Review Board (IRB) of Martinique University Hospital (IRB 2022/172). All participants gave their consent to participate to the study.

Descriptive statistics were obtained for all study variables. Continuous data are expressed as median and interquartile range. Categorical data are expressed as number and percentage.

## 3 Results

In total, 40 patients with MIS-C were hospitalized in FWI between April 1, 2020, and August 31, 2022. The first documented case of MIS-C admitted to PICU in the FWI was in January 2021. The FWI have a combined population of approximately 330 000 children [11,12], the incidence of MIS-C cases was 12 per 100 000 children. Twenty-three were girls (57.5 %), the median age was 7 years (IQR 5–11). The patient characteristics, clinical signs and symptoms, and laboratory values are detailed in Table 1. Only 6 (15 %) had comorbidities (asthma (n = 2), obesity (n = 3) and sickle cell disease (n = 1). Most of the patients (n = 31 (77 %)) were included prospectively. On admission, the median duration of fever was 5 days (IQR 4–6), 16 patients (40 %) were in shock and received vascular filling, and 30 (75 %) had decreased cardiac function (Table 1). No patient had initial neurological, kidney or liver failure. The link with SARS-CoV-2 infection was proven for each patient (4 positive nasal PCR alone, positive serology for the others). None of the children included was vaccinated against SARS-CoV-2, and only 2 of them had vaccinated parents.

**Table 1:** Patient's demographic and clinical characteristics on admission.

	Our study
	n = 40
Sex	
Male	17 (42 %)
**Age, years**	7 [5–11]
**Weight, kg**	25 (18–49)
**Department of origin**	
Martinique	19 (47 %)
Guadeloupe	15 (37 %)
French Guiana	4 (10 %)
Saint Martin	2 (5 %)
**Clinical signs and symptoms**	
Fever. Days Acute gastrointestinal symptoms Pharyngitis	5 [4–6] 35 (87 %)
	24 (60 %)
Conjunctivitis	20 (50 %)
Rash	18 (45 %)
Lymphadenopathy	13 (32 %)
**Laboratory values**	
Fibrinogen (g/L)	6.4 (4.1–8.1)
D-dimer (μg/mL)	3.6 (1.7–6.5)
Troponin (pg/mL)	69 (21–268)
BNP (pg/mL)	1593 (145–3825)
*C*-reactive protein (mg/L)	181 (140–240)
Ferritin (ng/mL)	942 (533–1923)
Platelets count (X10^9^/L)	179 (138–253)
Neutrophil count (X10^9^/L)	8.8 (6.8–14.4)
**Outcomes**	
Decreased cardiac function Shock Vasoactive and inotropic support Persistent fever 36 h after end of initial IV infusion Median length of intensive care stay (days) Death	30 (75 %) 16 (40 %) 18 (45 %) 8 (22 %) 5 [3–6] 0

Data are n (%) or median (IQR). BNP = brain natriuretic peptide. *Discharged from critical care at D4 74 (96 %). Laboratory's reference values for Fibrinogen 1.9–4.8 g/L D-dimer <0,5 μg/mL Troponin <33 pg/mL BNP>100 pg/mL CRP <5 mg/L Ferritin 22–275 ng/mL Platelets 150-300G/L Neutrophil count 4-10G/L.

**Fig. 1:**
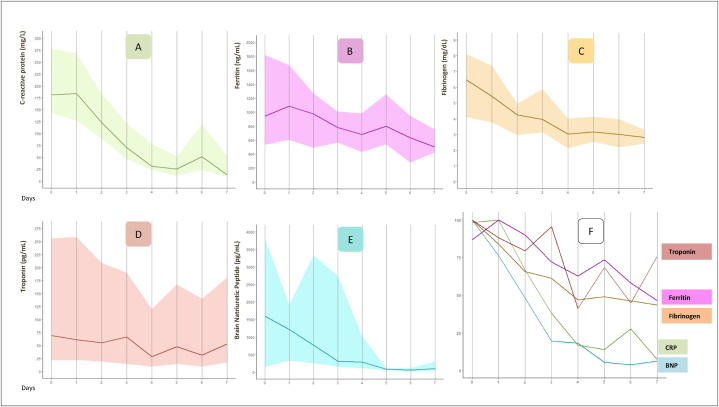
Laboratory markers of inflammation and cardiac enzyme for the 40 patients from day 0 (admission) to day 7, data are median for each day represented by curve, colored area represented interquartile range. A = curve of median values of *C*-reactive protein and interquartile range. B = curve of median values of Ferritin and interquartile range. C = curve of median values of Fibrinogen and interquartile range. D = curve of median values of Troponin and interquartile range. E = curve of median values of Brain Natriuretic Peptide and interquartile range. F = dynamic of all markers. Logiciel, package “ggplot2”.

The monitoring of inflammatory markers and cardiac dysfunction from day 0 to day 7 is illustrated in Fig. 1(A-F). Triglycerides levels were normal to subnormal for most patients at day 0 and day 7. No patient suffered from acute anemia from day 0–7. Platelet count progressed to moderate thrombocytosis at day 7 (530 G/L; IQR 273–656). Regarding cardiac markers (Fig. 1D–E), troponin levels were highly variable between patients at day 0 (range: 5–33 731 pg/mL) and only 35 % of patients had normal values at day 7. The curve of median BNP from day 0–7 is illustrated in Fig. 1E, median value at day 7 was subnormal, at 100 pg/mL (IQR 38–305 pg/mL), but this was not associated with persistent clinical cardiac dysfunction.

Cardiac dysfunction was maximal during the first three days after admission (Table 2). Initial cardiac dysfunction was both systolic (systematically restored at day 7) and diastolic (mostly persisting at day 7). Five patients were followed up at 3 months, all had LVEF>60 %, median IVRT was 95 m s and none showed signs of relapse or sequelae. Pericarditis was found in 14 patients (45 %) at day 0, in 7 (29 %) at day 3 and in 9 patients (26 %) at day 7. No patient had coronary dilation or aneurysm. Right ventricular function was subnormal at D0, 10 patients (27 %) had TAPSE < -2 standard deviations in age, then at D3 and D7 all of them had a normal TAPSE (Table 2).

**Table 2:** Ultrasound Data from day 0 to day 7.

	Cardiac Ultra Sound D0 (n = 36)	Cardiac Ultra Sound D3 (n = 26)	Cardiac Ultra Sound D7 (n = 34)
Left systolic function			
LVEF ≥60 %	6 (17 %)	13 (52 %)	30 (88 %)
45 % ≤ LVEF <60 %	13 (36 %)	10 (42 %)	3 (9 %)
30 % ≤ LVEF ≤44 %	15 (42 %)	1 (4 %)	1 (3 %)
30 % < LVEF STRAIN	2 (6 %) −9.5 (−16.2–−8)	0 −19 (−20.2–18.5)	0 −20 (−22.7–−18.20)
**Left diastolic function**			
IVRT, ms	66 (50–87)	65 (60–90)	72,5 (59–91)
E/E’	7.5 (6.6–8.5)	7 (6.5–9)	8 (6.5–9.5)
E/A	2.2 (1.4–2.6)	1.9 (1.6–2)	1.5 (1.4–1.9)
**Right function**			
TAPSE, Z-Score	−2,1 (−4,0–1,0)	0,8 (−0,4–1,6)	0,2 (−0,9–1,4)
Onde S, cm/s	11 [9–15]	11.5 [11,12]	12.5 [11–15]

Data are n (%) or median (IQR). D0 = on admission. LVEF = left ventricule ejection fraction, IVRT = isovolumic relaxation time, TAPSE = tricuspid annular plane systolic excursion, Z-Score (standard deviations in age) calculated on parameter(Z).

Inotropes were required in 17 patients (42 %) and the drugs and doses used are detailed in Table 3. Respiratory support by invasive and/or non-invasive mechanical ventilation was required in 7 patients (4 were intubated). No patient developed ventilator-associated pneumonia and no patient died. The most severe patient had acute respiratory distress syndrome and required extra corporeal membrane oxygenation. The median length of stay in PICU was 5 days (IQR 3–6; range 2–15 days). Only one patient was readmitted after initial discharge from the PICU due to persistent vasoplegia, which resolved spontaneously favorably.

**Table 3:** Amines and vasopressors’ Intensive care patients.

Inotropes (n = 40)	17 (42 %)
Time SOS* (days)	5 [5–7]
Duration (hours)	96 (24–98)
**Adrenaline**	1 (2.5 %)
Max dose (μg/kg/min)	0.3
**Dobutamine**	16 (40 %)
Max dose (μg/kg/min)	5 [5–10]
**Milrinone**	2 (5 %)
Max dose (μg/kg/min)	0.6 (0.5–0.8)
**Levosimendan**	1 (2.5 %)
Max dose (μg/kg/min)	0.2
**Vasopressors (n** = **40)**	
**Noradrenaline**	7 (17 %)
Time SOS* (days)	7 [5–8]
Max dose (μg/kg/min)	0.5 (0.1–0.7)
Duration (hours)	12 (4–72)

Data are n (%) or median (IQR). *Time SOS = Time since onset of symptoms.

The median time from the onset of symptoms to the first dose of treatment was 5 days (IQR 4–6 days) for steroids and 5.5 days (IQR 5–6 days) for IVIg. Fever persisted at 36 h of treatment in 8 patients (22 %); most of them responded well to a second IVIg course, and only 2 patients received biological therapy (anakinra). No patient suffered significant clinical, metabolic or biological side effects.

Since the differential diagnosis was bacterial infection, every patient received antibiotic therapy at onset and the median duration of treatment was 4 days (IQR 2–6). Healthcare-associated infections occurred in two patients: one urinary tract infection (wild-type *Klebsiella pneumoniae)* and one central line-associated catheter infection (*Staphylococcus aureus)*.

**Fig. 2:**
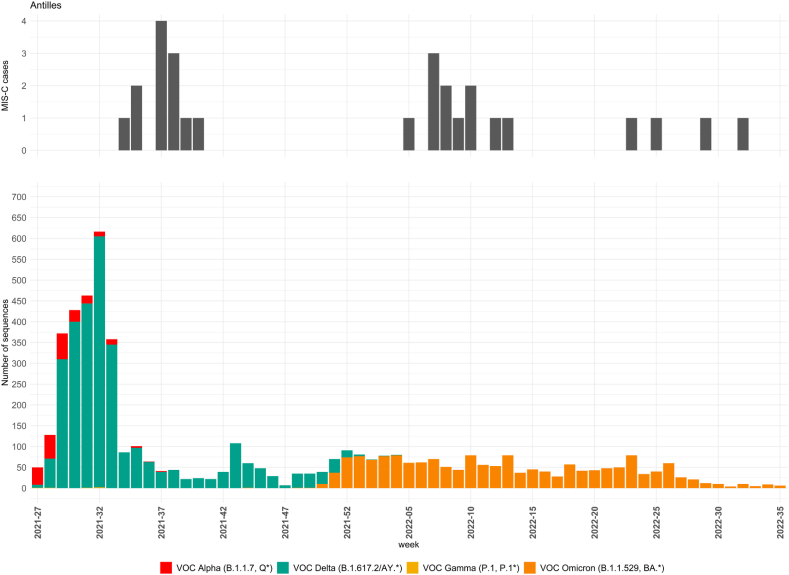
Cases of MIS-C and SARS-CoV-2 variants circulating in the FWI from 2021-Week27 to 2022-Week35. SARS-CoV-2 genomic surveillance was based on selected positive reverse transcription followed by quantitative polymerase chain reaction (RT-qPCR) tests.

The distribution of cases of MIS-C and SARS-CoV-2 variants over time is shown in Fig. 2. The FWI were hit by the fourth French epidemic wave mostly caused by the Delta variant [13]. Thirteen patients with MIS-C (33 %) were included during this epidemic wave. The Delta variant wave lasted from 14 to 18 weeks in total, with a peak incidence in Guadeloupe of 2287/100 000 (1196 in Martinique and 542 in French Guiana compared to 248/100 000 in mainland France). The following epidemic wave was mainly related to the Omicron variant, and lasted longer, namely 25–34 weeks, with a peak incidence in Guadeloupe of 4592/100 000 (3822 in French Guiana, 3759 in mainland France and 3206 in Martinique). We observed 15 cases of MIS-C (50 %) during these 34 epidemic weeks. Finally, 12 cases of MIS-C were retrospectively identified from the first three epidemic waves beginning in week 11 of 2020 in FWI, the dominant variant identified being Alpha in Martinique and Guadeloupe at that time. The incidence curves show that most MIS-C cases appeared and followed the peak in the number of cases in the population with a 4–6-week lag time. In addition, the vast majority of cases of MIS-C were recorded within 6 weeks after exposure to a new SARS-CoV-2 variant.

## 4 Discussion

This ambidirectional multicenter study is the first report of a pediatric cohort of Afro-Caribbean children, and it supports current knowledge about MIS-C in terms of patient characteristics, clinical features and favorable outcomes [1]. No other reports to date have provided such a detailed, day-by-day description of the laboratory tests and echo results dynamics in a population of Afro-Caribbean children with MIS-C.

Gastrointestinal symptoms were major clinical features in our cohort as previously described [1,3,9,14,15]. Our patients with MIS-C presented few cutaneous or ear/nose/throat (ENT) symptoms, which differs from the classical description of Kawasaki disease (KD). KD predominantly affects young children under the age of 5, whereas the median age in our cohort was 7 years, and no child in our series was aged under 5. The median age of patients in our study was numerically lower than in other reports, which may have impacted the severity of disease, although the age range of affected patients is coherent with other series [9,14,[16], [17], [18]]. The majority of patients (57.5 %) were girls in our study, which is contrast with other reports, where a predominance of boys was reported [3,9]. Left ventricular dysfunction was the main cardiac feature in this series, with no patient having coronary artery dilation. Today, the definition of MIS-C is more precise, and we did not capture atypical presentations in our cohort.

Regarding biological features, troponin values were highly heterogeneous in our patients, but even patients with very high values quickly recovered normal systolic function. Some reports showed that BNP levels were very high, whereas troponin elevation was usually mild to moderate [3,19], which was more or less in line with the observations in our cohort, apart from a few extreme cases. A meta-analysis reported that BNP levels were higher in patients with severe MIS-C than in those with non-severe MIS-C [20]. In our study, treatment was not modified on the basis of changes in biological markers alone, but rather, was only changed in case of the dual presence of both a clinical deterioration, and corresponding changes in the biological results. Thanks to the repeated lab tests, we were able to monitor the course of biological markers on a day-to-day basis. The literature suggests that cardiac markers may be monitored carefully at different intervals in patients admitted with MIS-C, to predict potential deterioration during the disease course [[20], [21], [22]]. Our dynamic monitoring of biological inflammatory and cardiac markers showed that the decreasing slope of the curves matched after treatment, and their levels were not associated with acute dysfunction or further sequelae. Further studies are needed to evaluate the benefit of cardiac markers monitoring in medical care and prognosis of MIS-C.

The clinical and biological features of our cohort are comparable to the classic description of MIS-C [1,3,9,14,15]; namely, severe disease marked by an inflammatory storm causing organ dysfunction, with cardiogenic shock in 40 % of cases and vasoactive and inotropic support required in 45 % of cases. Early etiological treatment (steroids and IVIg) was mostly successful in treating vasoplegia. The outcomes in our population were favorable, with no deaths, and rapid recovery of cardiac function, as described in the literature [1,3,[21], [22], [23], [24]]. Thanks to the experience of previous epidemic waves in western countries, we chose to admit most of the patients to PICU from the time of the first vascular filling, hypothesizing that early active treatment (vasoactive and inotropic support) in addition to etiological treatment could reduce the length of stay, avoid strain on hospital resources, and avoid the need for *trans*-Atlantic medical evacuation. There is no high-level scientific evidence to date regarding the benefit of early treatment of MIS-C in terms of cardiac injury and the risk of sequelae. However, our patients all received prompt etiological treatment (as soon as they met the definition of MIS-C) and had excellent overall prognosis. The most severe patient who required extra corporeal membrane oxygenation had 36 h delay in etiological treatment which, in our opinion, may explain the severe phenotype.

Black or Afro-Caribbean ethnic origin has been shown to be related to severe forms of MIS-C [3,9,15,18,25]. The French healthcare system is universal and free, thus the bias related to socioeconomic status and access to healthcare is likely to be less impactful in the French overseas Departments, and the outcomes of our patients are more in favor of socio-economic factors worsening the prognosis, rather than any ethnic susceptibility. This has already been shown for other auto-immune or inflammatory diseases in our pediatric population [11,12]. The number of MIS-C cases observed in Martinique, Guadeloupe and French Guiana was 40 over a 2.5-year period (incidence of 12 cases per 100 000 children). The total in France was 1092 cases of MIS-C (with an incidence in mainland France of 7,5 per 100 000 children), 68 % required hospitalization in the PICU [26]. MIS-C incidence in FWI was higher than mainland France, partially explained by SARS-CoV-2 incidence peak much higher in FWI than in mainland France (almost 10 times for Delta variant). Overall, our work does support an ethnic susceptibility to increased incidence but not to increase severity of MIS-C in an Afro-Caribbean population. Our data support the overall pattern of MIS-C onset, which usually occurs approximately 4–6 weeks after SARS-CoV-2 infections and hospitalizations in the general population [1,3,7,14,27]. Our results about the ratio of MIS-C incidence over total cases of SARS-CoV-2 suggests that the Omicron variant is less prone to induce MIS-C than the Delta variant. This has previously been reported in Western countries [28,29].

A large vaccination campaign began in France in December 2020 in the general population and as of June 2021, was also offered to children aged 12 years or over. The vaccination campaign was then extended to all children from age 5 and upwards in December 2021. Adult vaccination rates in the French overseas Departments were among the lowest in all of France, therefore we were not able to study impact on vaccination on MIS-C incidence. No child in our cohort was vaccinated, and only 2 patients (5 %) had parents who were vaccinated. The contribution of vaccination to MIS-C is unknown, but some studies suggest that MIS-C after COVID-19 vaccination is rare [30,31].

The protocolized biological and echocardiographic investigations in our patients found early systolic and diastolic dysfunction with rapid recovery of cardiac function and markers. These findings strongly differ from other acute myocarditis in children. Systolic dysfunction was measured by LV strain on 2D ultrasound and the conventional Teichholz method, both matched. From day 3, systolic function was partially restored as previously described [32]. Matsubara et al. suggested that 2D LV strain can detect abnormalities in cardiac function that go unrecognized by conventional echocardiography [21], but strain assessment requires sophisticated ultrasound machines and operator know-how that most pediatric intensivists do not possess. Diastolic dysfunction in our study was defined as IVRT<90 m s and might be the most accurate parameter for monitoring diastolic dysfunction as already described in MIS-C patients. In the study by Belhadjer et al. [32], the median time to recovery of IVRT>90 m s was 7 days, whereas in our cohort median IVRT on day 7 was 72.5 m s.

### 4.1 Study strengths and limitations

This study has several key strengths, namely it is the first description of the disease in an ethnic group that is under-represented in current literature, and it provides detailed data on the dynamics of the clinical course. Conversely, this study also has some limitations, including the small sample size. Second, part of the data collection was retrospective (12 of the 40 cases). However, the major epidemic waves in the French overseas Department were from 2021-W26 onwards, and the majority of cases (70 %) were included prospectively. Third, due to the severity of the disease and the geographic situation with only one PICU for the whole French Caribbean region, we believe that no cases of MIS-C patients were overlooked and that our cohort of FWI children is thus exhaustive. However, we cannot rule out the possibility that some cases were not diagnosed. Fourth, we included only patients who were admitted to PICU, and thus the results may not be generalizable to less severe forms of the disease. Fifth, the low number of MIS-C in our population may also be partially explained by the low impact of the alpha variant in terms of number of infections in the FWI. Finally, the absence of a control group of children representing different ethnic groups precludes any comparison of the severity of disease with children of other ethnic origins. Similarly, the fact that we chose to admit all children with MIS-C to PICU means that our results may not be generalizable to populations treated outside the PICU.

## 5 Conclusion

This ambidirectional cohort of MIS-C is the first report among children of Afro-Caribbean descent. We describe clinical, biological and echocardiographic characteristics at onset and during PICU hospitalization. Overall, outcomes were favorable, with no deaths, and with rapid resolution of abnormalities in cardiac function.

## Data availability statement

Data will be made available on request to the corresponding author.

***Ethics approval*** The study was approved by ethic committee of the CHU de Martinique.

***Consent to participate*** All participants have given their consent to participate and publication.

***Availability of data and materials*** All data generated or analyzed during this study are included in this published article and tables.

## Funding

N.A.Our work did not get any financial assistance and was not submitted to any other journal.

The study data were not presented or in consideration pour publication elsewhere.

We thank Julie D'ORAZIO and Alix DE GONNEVILLE for their assistance in performing cardiac echocardiography.

## Key points

**Question:** What are the characteristics and outcomes of multisystem inflammatory syndrome in children of Afro-Caribbean descent?

**Findings:** In this ambidirectional observational cohort that included 40 children, mostly prospectively, the clinical features and the outcomes of Afro-Caribbean children with MIS-C were similar to western countries without any death or sequelae.

**Meaning:** Our work does not support an ethnic susceptibility for severity of MIS-C in an Afro-Caribbean population.

## CRediT authorship contribution statement

**Charlène Grabot:** Writing – review & editing, Writing – original draft, Methodology, Investigation, Conceptualization. **Mélanie Brard:** Writing – review & editing, Investigation. **Daphnée Hilaire:** Writing – review & editing, Investigation. **Moustapha Drame:** Writing – review & editing. **Gwladys Nadia Gbaguidi:** Writing – review & editing, Formal analysis. **Narcisse Elenga:** Writing – review & editing. **Saskia Tuttle:** Writing – review & editing, Investigation. **Yves Hatchuel:** Writing – review & editing, Investigation. **Michaël Levy:** Writing – review & editing, Methodology. **Olivier Flechelles:** Writing – review & editing, Investigation, Conceptualization. **Arthur Felix:** Writing – review & editing, Writing – original draft, Validation, Supervision, Project administration, Methodology, Investigation, Formal analysis, Conceptualization.

## Declaration of competing interest

The authors declare that they have no known competing financial interests or personal relationships that could have appeared to influence the work reported in this paper.
