# Supplementary material for: Description and outcomes of Afro-Caribbean children treated for multisystem inflammatory syndrome in the French West Indies
Source: Heliyon. 2023 Nov 19;9(12):e22642. doi: 10.1016/j.heliyon.2023.e22642 (PMC10687232; doi:10.1016/j.heliyon.2023.e22642)
Supplement: Multimedia component 1 [file mmc1.docx]

**DESCRIPTION AND OUTCOMES OF AFRO-CARIBBEAN CHILDREN TREATED FOR MULTISYSTEM INFLAMMATORY SYNDROME IN THE FRENCH WEST INDIES**

**Figures supplementary data: 1**


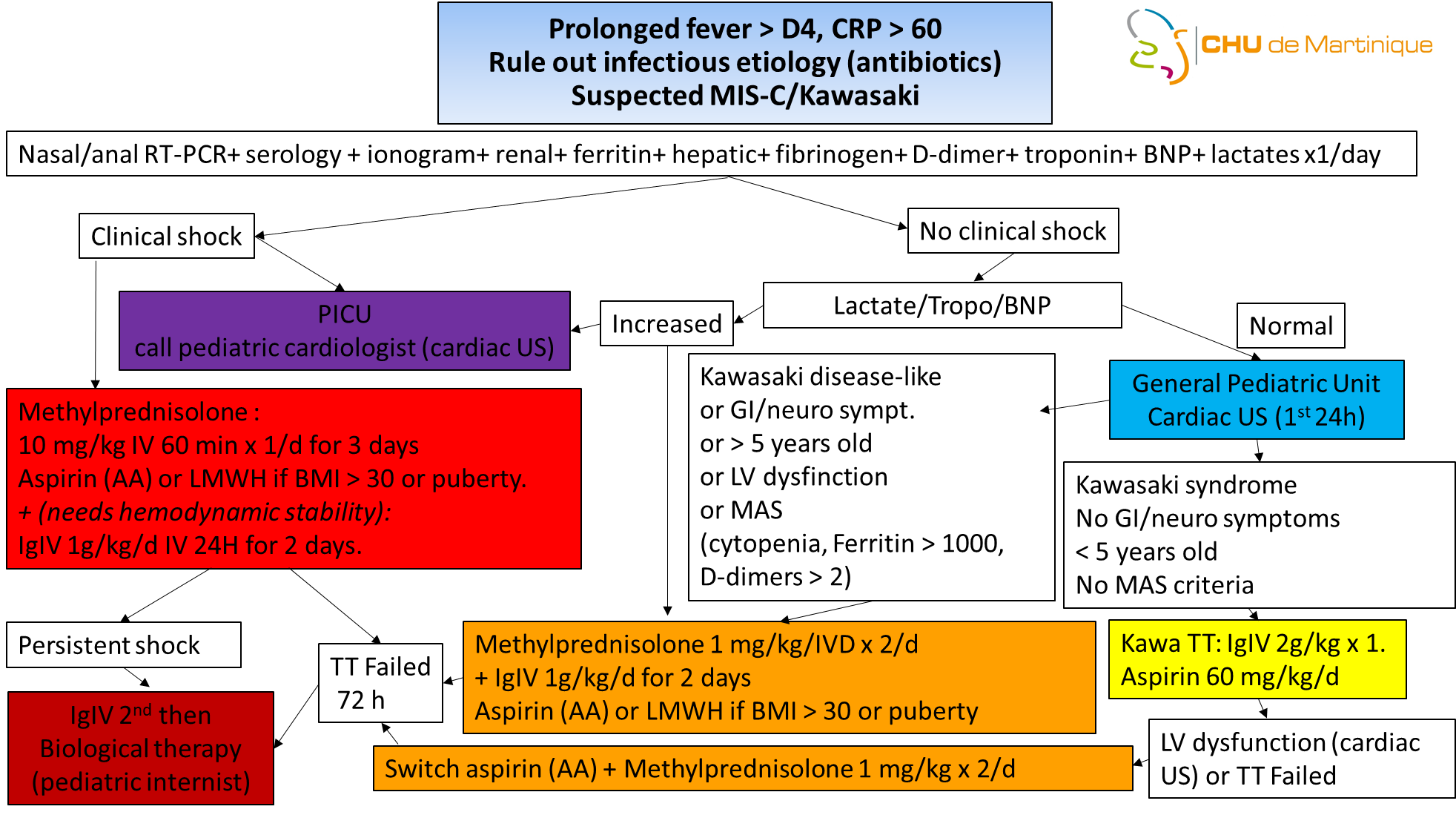


**Supplementary Figure 1: Treatment protocol**

*D=Days. Cardiac US= cardiac Ultrasound. Aspirin (AA)= Anti-aggregation Aspirin. LMWH=Low Molecular Weight Heparin. GI/neuro sympt.=gastrointestinal and neurological symptoms. LV = left ventricular. MAS=macrophagic activation syndrome. IgIV=polyvalent immunoglobulins.*
